# Surviving, Learning, and Striving in the Times of Pandemic: Teaching With A Journal of the Plague Year: An Archive of COVID-19 (JOTPY)

**DOI:** 10.1177/1550190620981031

**Published:** 2021-09

**Authors:** Cheryl Jiménez Frei, Shane Carlson

**Affiliations:** 1 University of Wisconsin Eau Claire, USA

**Keywords:** archives, subject focus, digital collections, case study, teaching, rapid response collection, COVID-19 archives

## Abstract

With the onset of COVID-19, spring 2020 proved difficult for teachers and students everywhere. But amid the challenges of online and hybrid education, incorporating *A Journal of the Plague Year: a COVID-19 Archive* (JOTPY) into classrooms provided students a unique and impactful learning experience, while also helping them process the anxieties and uncertainties of the pandemic. In this article, Assistant Professor of History at the University of Wisconsin-Eau Claire (UWEC) Cheryl Jiménez Frei shares insights and best practices for teaching with JOTPY, and a model incorporating the archive across multidisciplinary courses to address archival silences. Beyond the university, JOTPY can be a valuable pedagogical tool for elementary, middle, and high-school teachers during the pandemic. To examine this, in the article’s second half, UWEC public history graduate student and high-school teacher for the Eau Claire Area School District Shane Carlson shares his reflections on contributing to the archive as a student, strategies bringing JOTPY into his own teaching, and the results of elementary teachers also doing so in rural Wisconsin.

As is common with consequential historical moments, most of us likely have our COVID “flashbulb” memory: vividly recalling the moment we realized the risks of COVID-19, and how it would alter daily life in ways we never imagined. For those of us in education—both teachers and students—that moment may have come when classes moved online in spring 2020. As teachers rushed to re-work courses, retain learning outcomes and consider students’ varying circumstances, students struggled to process expectations and lost opportunities in the unpredictable weeks ahead.

Needless to say, spring 2020 proved a challenge for teachers and students everywhere. With the pandemic continuing and most institutions adopting fully virtual or hybrid models combining in-person and online elements for the full 2020–2021 academic year, COVID-19 remains a part of our everyday landscapes. In these times of uncertainty and adaptation, how can educators provide impactful learning experiences while helping students process the pandemic? Incorporating *A Journal of the Plague Year: a COVID-19 Archive* (JOTPY) into teaching provides an answer. In this article, we share our experiences and suggestions from our relevant perspectives: those of a university professor, a graduate student, and a high-school teacher. Overall, our insights provide best practices on teaching the archive in university courses; a model for a collaborative, multidisciplinary learning experience incorporating JOTPY to address archival silences; and strategies for utilizing JOTPY as a valuable pedagogical tool in K-12 classrooms.

## Multi-disciplinary Engagement, Archival Silences, and Connecting Past to Present

### JOTPY in University Classrooms, Cheryl Jiménez Frei

When the pandemic first hit, I found myself facing a situation similar to many other public history faculty: with students immersed in a field project that simply could not translate online. My undergraduate and graduate students had spent weeks preparing an exhibit for a local welcome center, so the decision to drop the project was a difficult one. Public history by nature is hands-on, and it is key—especially in an upper-division seminar, which I was teaching—for students to gain experience in the field. Brainstorming options, my thoughts remained in our current moment and the many rapid-response collection efforts that were beginning to take shape. How will COVID-19 be remembered? In a pandemic with global reach, whose stories will be preserved?

**Figure 1.**
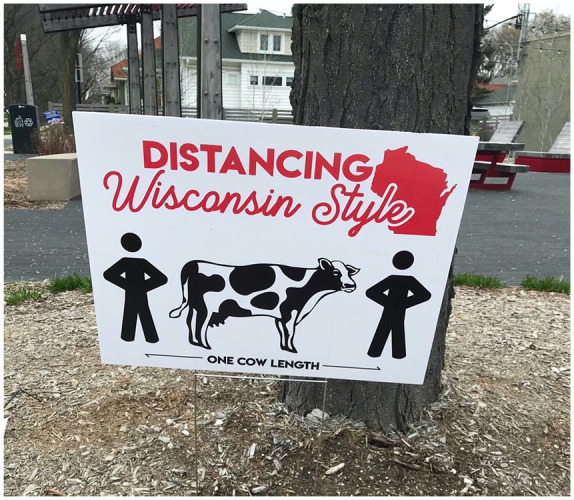
A sign in Milwaukee uses Wisconsin’s dairy industry to provide a relatable reference for social distancing. Photo submitted by UWEC graduate student Nick Eggert, April 29, 2020. *Source*. https://covid-19archive.org/s/archive/item/31745.

**Figure 2.**
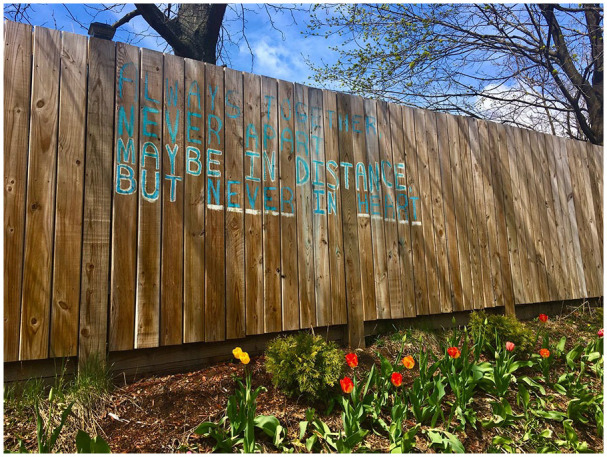
A chalk drawing on a neighborhood fence in Chippewa Falls, Wisconsin reflects distancing measures with the words: “always together, never apart; maybe in distance, but never in heart.” Photo submitted by UWEC graduate student Shane Carlson, April 29, 2020. *Source*. https://covid-19archive.org/s/archive/item/31747.

I posed students the choice: continue a limited, digital version of their previous project, or shift completely to documenting the history of the pandemic and its effects in our communities for JOTPY. To their immense credit, they choose the latter. After leaving campus for towns across Wisconsin, Illinois, Minnesota, and South Dakota, the students recorded oral histories and collected artifacts for JOTPY, documenting experiences of the pandemic in the rural Midwest (see Figures 1 and 2).

Many students did not have oral history experience, but running their first interview with family or friends was a good strategy to acclimate. Overall, the perspectives they collected paint a wide picture of COVID-19’s effects across communities in the Midwest: from farmers struggling with a pandemic-related breakdown in food supply chains, to interviewees frustrated by protests of safety measures. These oral histories seemed unique, with interviewers living through the same circumstances as their interviewees—this lent empathy and connection, seeming to ease student interviewers into thoughtful follow-up questions. Surprisingly, students noted that conducting interviews over Zoom (a necessity that exemplifies changes brought by the pandemic) made the process easier.

JOTPY seemed an obvious fit for public history students, providing them an opportunity to respond to history as it happened, while practicing skills in the field. As one of my graduate students said, “it seems like our duty as historians to document what is happening.” Others felt it allowed them to do something meaningful in a chaotic moment. Inspired, I brought JOTPY into another course in spring 2020, a Latin American history survey, a course where I realized the primarily non-history majors enrolled were still struggling with using primary sources to understand the past. JOTPY seemed an ideal solution, and one also providing students an outlet to process swirling emotions.

To facilitate this, a colleague and I developed an assignment: “Documenting Your Experiences: Creating a Primary Source”, which is currently being adapted by local high-school teachers. It’s designed in a vein of an “un-essay,” encouraging students to creatively interact with class themes and outcomes by allowing *them to choose the medium* to present their ideas. Students had expressive freedom, and were asked to donate sources to JOTPY. They also wrote reflections on historical methods learned in the course and what future researchers could learn from their primary source.

**Figure 3.**
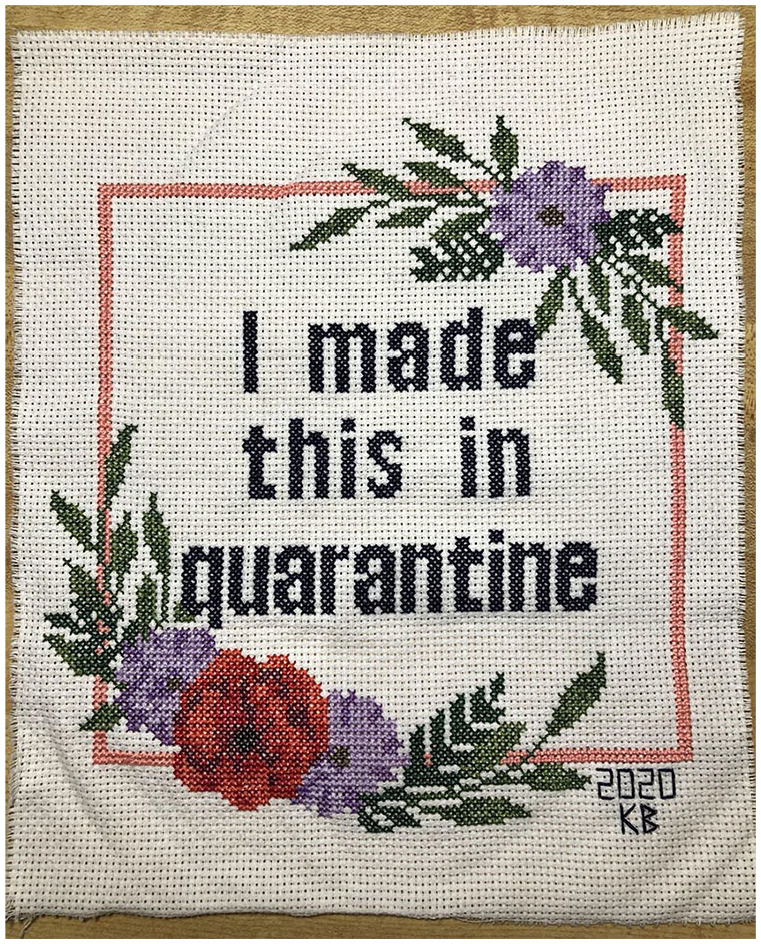
A cross stitch created and donated to JOTPY by undergraduate Katie Boucher, May 7, 2020. *Source*. https://covid-19archive.org/s/archive/item/31751.

**Figure 4.**
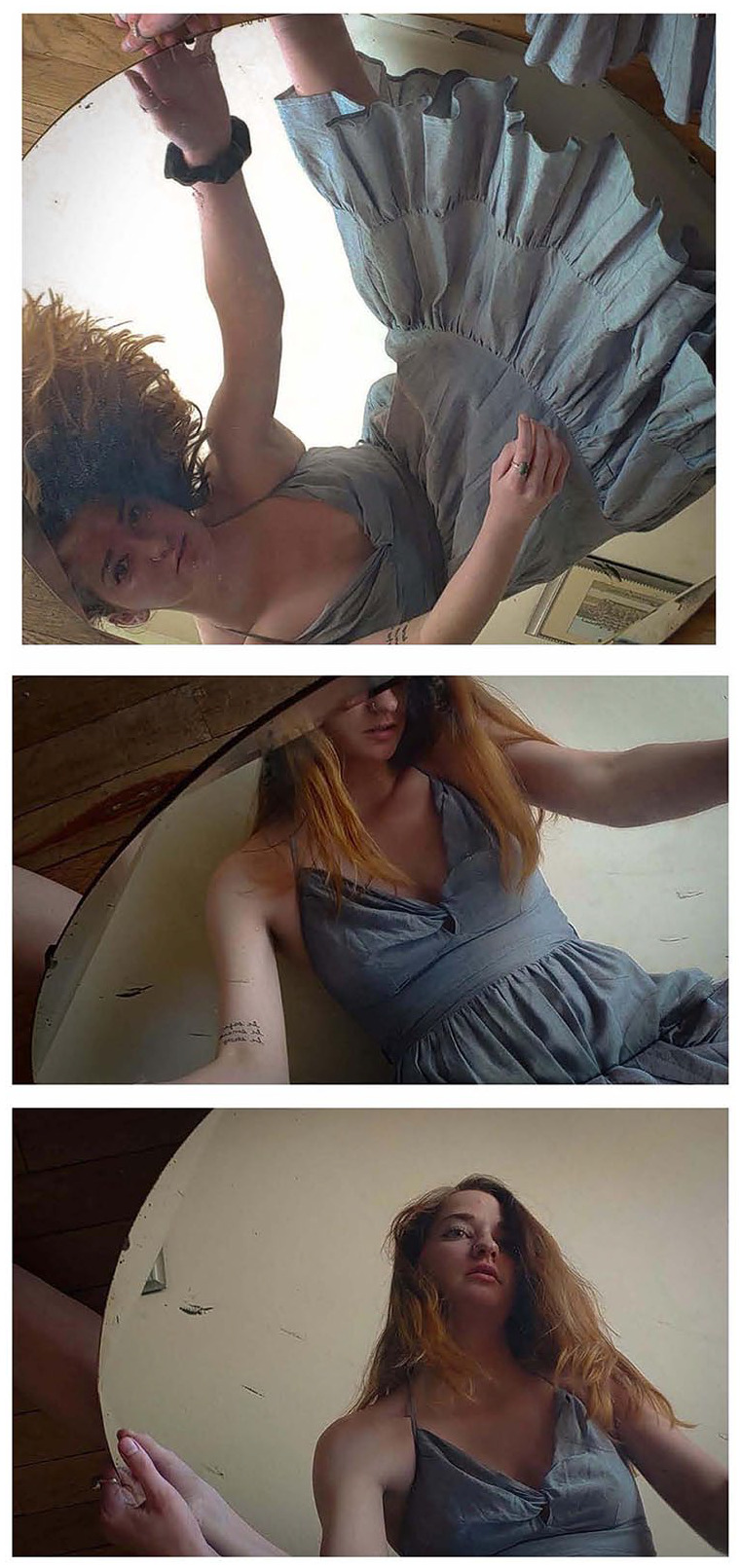
For this anonymous submission, the student explained their self-portraits as expressing disorientation and self-reflection spurred by quarantine, using a mirror to “reflect the immense amount of time I’m stuck in my own head recently.” Photos submitted anonymously, May 20, 2020. *Source.*https://covid-19archive.org/s/archive/item/24904.

Students responded in innovative ways, creating poetry, comics, short films, cross-stitches (Figure 3), photo and written journals, and even expressed the rollercoaster of their experiences through piano arrangements. Some expressed painful experiences with depression, anxiety, or unstable living situations, and choose not to donate these items, or did so anonymously (Figure 4).

Overall, assigning JOTPY allowed students to better understand the nature and purposes of archives, and the importance of primary sources to history work. It also helped them process emotions in uncertain times, and their artifacts drew empathetic connections at a time when we were all distant. Many thanked me for incorporating JOTPY, and the materials they produced proved profound and insightful.

**Figure 5.**
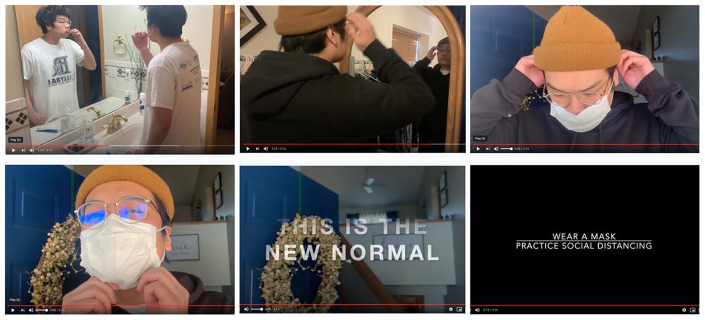
Stills from a public service announcement styled film donated by undergraduate Jaymar Basilio, May 21, 2020. *Source.*https://covid-19archive.org/s/archive/item/31755.

Also significant was the students’ documentation of the pandemic’s outset, when many rural areas did not yet see a rise in cases. For many, COVID-19 seemed distant, with safety measures disregarded. One undergraduate choose to create a public service announcement, filming his brother’s altered morning routine to encourage others to follow regulations (Figure 5). In his reflection, he wrote:

*In Minnesota, confirmed cases are not as extreme as in other states like New York. . . On social media, many of my peers are complaining about when [the restrictions] will be over. People are going out acting like life is normal, hanging with friends without social distancing and not wearing masks. . . . but the consequences are increasing the chance of the virus spreading. I have uncles and aunts that . . .are at a higher risk by the coronavirus so I grocery shop for them every week. When I go to grocery stores, I get very anxious because many people are not wearing masks. I don’t want to be the one responsible for getting corona and getting my family sick.*

With students back on UWEC’s campus in fall 2020, cases have risen and daily awareness of COVID-19 has shifted. I anticipate students’ documentations will reflect this, and the archive will again provide a powerful learning experience while helping students process anxieties surrounding the pandemic.

Students in my world history surveys will also use JOTPY to research, in an assignment asking them to analyze/compare sources on the Black Plague, 1918 pandemic, and from JOTPY. Here I utilized open-access JOTPY teaching modules,^1^ which provide insightful tools including readings, discussion questions and activities, using JOTPY to teach about digital archives, rapid-response collection, and archival silences.

With 2020’s protests against racial inequality, students are increasingly aware of systemic racism in the U.S.—something COVID-19 has brought into even starker relief. For educators, this moment should inspire critical thinking. In my own courses, this means questioning *how* history is told—*whose* voices have been absent, and *what* role do archives play? Students in my world history courses will explore these questions using JOTPY, as they research to examine archives’ civic purposes and silences, during pandemics both past and present.

The issue of archival silences also inspired shifts for students collecting oral histories in my public history courses. In the spring, despite shared goals seeking voices from Asian, Latinx, Indigenous, and Black communities—marginalized in rural areas and the imaginary of the American Midwest—students gravitated towards interviewees who looked like them: this meant majority white. As I again incorporate JOTPY, students must interview at least one person outside their own community, to address silences while spurring discussions of archives and power. Their work with JOTPY will also involve discussions and considerations for rapid-response collection and digital archiving.

Lastly, I teamed with colleagues on a multi-disciplinary project utilizing JOTPY to document experiences of immigrant farmworkers during the pandemic. Our project, titled *Documenting the Undocumented*, joins faculty and students in Public History, Spanish, and Nursing to collect oral histories with Spanish-speaking immigrant and undocumented workers in western Wisconsin.

Through our collaboration, nursing students enrolled in UWEC’s *Health Care for Immigrant and Local Farmers Clinical Immersion Program*—which brings nursing students to rural farms, providing health screenings and immunizations for immigrant workers—will conduct Spanish-language oral histories with their patients. Students in a *Spanish for Health Professions* course will also record, transcribe, and translate interviews with workers, while public history graduate students will process and digitally archive these materials for JOTPY. This multi-disciplinary project, directly inspired by incorporating JOTPY into our diverse classrooms and field work, provides innovative learning experiences across the humanities and sciences, and is one I hope may serve as a model for future projects documenting rural and often silenced voices, here at UWEC and other universities in the Midwest.

## Finding Relevance, Fostering Empathy, and Connecting Communities

### JOTPY in K-12 Classrooms, Shane Carlson

During the early stages of the pandemic, I was among the students in Dr. Jiménez-Frei’s public history seminar, gathering oral histories and artifacts to document the public health crisis. As a graduate student contributing to JOTPY, COVID-19 became a call to service and an opportunity to rethink educational approaches. Conducting oral histories in my community turned out to be surprisingly cathartic, and commiserating with fellow graduate students helped soften the abrupt shifts, providing reassurance that I was not alone.

However, my experiences as a high-school teacher proved more challenging. Reduced curricular expectations could hardly alleviate the shock to student well-being and academic development. Like so many educators, I had to rethink student engagement through virtual platforms. I worried about how to meet students’ social-emotional needs during times of crisis while providing meaningful learning experiences. Fresh from reading Nina Simon’s *The Art of Relevance* and in the midst of conducting oral histories for JOTPY, I began to strategize how to incorporate the archive into my own teaching, and to make this a relevant learning experience for students.

As in university courses, utilizing archival collections helps introduce middle and high-school students to primary sources and contextual evidence. Here in Eau Claire, fifth-grade teachers at Sherman Elementary School had students engage with primary sources by having them document experiences of the pandemic in a journal and donate these to JOTPY. Teachers provided prompts asking students to reflect on ways adults talked about COVID-19, differences in life before and during the pandemic, and what they would do once things return to normal. Journaling was used in conjunction with Lauren Tarshis’s *I Survived* Scholastic graphic novel series, which “tells stories of young people and their resilience and strength in the midst of unimaginable disasters.”^2^

**Figure 6.**
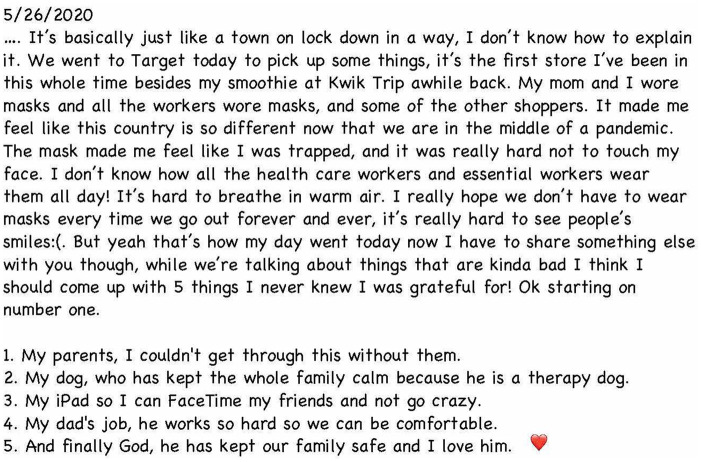
Journal excerpts by fifth-grader Ella Riechers at Sherman Elementary, describing her experiences with COVID-19. *Source.*https://covid-19archive.org/s/archive/item/31758.

The elementary school project made its mark in the classroom, where it served as a literacy builder for the social sciences. Students used new terminology demonstrating fluency and comprehension of lived experiences across physical and digital mediums. The project also encouraged students to empathize with each other and relate to young persons living through past traumatic events, laying the groundwork for discussions of historical perspectives. According to one teacher implementing the project, it was especially helpful in monitoring students’ welfare, as they catalogued thoughts and feelings. Many parents chose to donate student’s reflections to JOTPY (Figure 6). Further efforts bringing the archive into elementary classrooms will allow students to explore personal experiences of the pandemic across the globe.

A realistic addition to the high-school classroom includes analyzing parallels of the U.S. pandemic response between sources from Nancy Bristow’s *American Pandemic* and JOTPY. *American Pandemic* documents the U.S. response to the 1918 pandemic, silenced narratives, and gendered understandings that carry several correlations with COVID-19. Bristow used archival evidence to describe gendered reactions to the pandemic: while women often detailed personal grief, helplessness, and concerns for their communities, male counterparts projected “an image of masculine detachment,” through journals and correspondence. In my own classroom, I will also incorporate the University of Leeds project, *Using Archives to Teach Gender* (which provides access to 150 artifacts and documents that relate to gendered and feminist interpretations of the past), to allow students to explore different ways history can be gendered.

Educators seeking to go beyond exploring the archives have a unique chance to transform the history classroom by having their students become contributors to JOTPY. The Society of American Archivists’ *Documenting in Times of Crisis: A Resource Kit* can be helpful for educators seeking to do this, alongside K-12 modifications to JOTPY teaching modules.^3^ JOTPY’s response collection effort welcomes student participants to perceive the fleeting present in their communities as an approaching past.

The most compelling reason to use JOTPY in middle and high school classrooms deals with how and why people engage with the past. Roy Rozenzwig and David Thelen sought to answer these questions in their infamous 1994 survey, which found that “[i]ndividuals turn to their personal experiences to grapple with questions about where they come from and where they are heading, who they are and how they want to be remembered.”^4^ For many, personal experiences hold the key to engagement with the past. Teachers don’t need to mask learning when students understand the content is relevant, and student-archivists may be too engrossed in memorable and meaningful work to realize they are acquiring lifelong critical thinking skills.

Ultimately, archival projects can also connect community members and help public institutions serve constituents—two things that in these times of isolation seem increasingly important. In planning possibilities to integrate JOTPY into teaching, I am reminded of a project titled “Hear Hear” by UW-LaCrosse, which recorded oral histories of LaCrosse, Wisconsin, and has since created correlated curriculum for grades 4, 8, and 9–12, further expanding opportunities for local community engagement. JOTPY carries similar potential to connect students, educators, public institutions, and community members in an effort to capture local experiences during an unprecedented time.

## Conclusion

As these experiences, reflections, and strategies reveal, JOTPY offers myriad ways to provide inventive and significant learning experiences for students: challenging them to think critically about the past and present, how we understand history, whose stories have been silenced, while also providing a venue to process experiences and participate in documenting history in real time. We hope that other educators will consider incorporating JOTPY into their classrooms, bringing this valuable resource to students while helping grow the collections to preserve a diverse picture of the pandemic’s effects on individuals and communities across the globe.

## Footnotes

1. Created by Northeastern University’s Victoria Cain and Jim McGrath, and Boston Public School teacher Claire Tratnyek for university courses. The curriculum, funded by the New England Humanities Consortium, is available on JOTPY’s website: https://covid-19archive.org/s/teaching/page/jotpy-teaching-modules-northeastern-university-funded-by-new-england-humanities-consortium.

2. Scholastic Inc. *I Survived*, available at: https://kids.scholastic.com/kids/books/i-survived/ (accessed 21 August 2020).

3. “Documenting in Times of Crisis: A Resource Kit,” Society of American Archivists, last modified September 5, 2019, available at: https://www2.archivists.org/advocacy/documenting-in-times-of-crisis-a-resource-kit.

4. Roy Rozenzweig and David Thelen, *Presence of the Past* (New York: Columbia University Press, 1998), 12.

## References

[bibr1-1550190620981031] Basilio Jaymar. “Covid Morning Routine.” Rural Voices Collection, A Journal of the Plague Year: An Archive of COVID-19, May 5, 2020. https://covid-19archive.org/s/archive/item/31755.

[bibr2-1550190620981031] Boucher Katie. “Quarantine Cross Stitch.” Rural Voices Collection, A Journal of the Plague Year: An Archive of COVID-19, May 7, 2020. https://covid-19archive.org/s/archive/item/31751.

[bibr3-1550190620981031] Bristow Nancy K. American Pandemic. New York: Oxford University Press, 2017.

[bibr4-1550190620981031] Carlson Shane. “Neighborhood Fence, Chippewa Falls WI.” Rural Voices Collection, A Journal of the Plague Year: An Archive of COVID-19, April 29, 2020. https://covid-19archive.org/s/archive/item/31747.

[bibr5-1550190620981031] Eggert Nick. “Social Distancing, Wisconsin Style.” Rural Voices Collection, A Journal of the Plague Year: An Archive of COVID-19, April 29, 2020. https://covid-19archive.org/s/archive/item/31745.

[bibr6-1550190620981031] Riechers Ella. “I Survived Covid-19.” Rural Voices Collection, A Journal of the Plague Year: An Archive of COVID-19, June 12, 2020. https://covid-19archive.org/s/archive/item/31758.

[bibr7-1550190620981031] Rozenzweig Roy Thelen David. Presence of the Past: Popular Uses of History in American Life. New York: Columbia University Press, 1998.

[bibr8-1550190620981031] Scholastic Inc. “I survived: When Disaster Strikes, Heroes are Made.” Accessed August 21, 2020. https://kids.scholastic.com/kids/books/i-survived/.

[bibr9-1550190620981031] Society of American Archivists. “Documenting in Times of Crisis: A Resource Kit.” Last modified September 5, 2019. https://www2.archivists.org/advocacy/documenting-in-times-of-crisis-a-resource-kit.

[bibr10-1550190620981031] University of Leeds. “Using Archives to Teach Gender.” Accessed August 14, 2020. https://gender-archives.leeds.ac.uk/.

